# New records for the shallow-water chiton fauna (Mollusca, Polyplacophora) of the Azores (NE Atlantic)

**DOI:** 10.3897/zookeys.312.4768

**Published:** 2013-06-24

**Authors:** Sérgio P. Ávila, Julia Sigwart

**Affiliations:** 1 Faculdade de Ciências da Universidade do Porto Rua do Campo Alegre s/n, Porto; 2 CIBIO, Centro de Investigação em Biodiversidade e Recursos Genéticos, InBIO Laboratório Associado, Pólo dos Açores, Açores, Portugal; 3 Departamento de Biologia, Universidade dos Açores, 9501-801 Ponta Delgada, Açores, Portugal; 4 Queen’s University Belfast, School of Biological Sciences, Marine Laboratory, Portaferry, BT22 1PF, Northern Ireland

**Keywords:** Azores, Mollusca, Polyplacophora, biodiversity, checklist

## Abstract

Published records, original data from recent field work on all of the islands of the Azores (NE Atlantic), and a revision of the entire mollusc collection deposited in the Department of Biology of the University of the Azores (DBUA) were used to compile a checklist of the shallow-water Polyplacophora of the Azores. *Lepidochitona* cf. *canariensis* and *Tonicella rubra* are reported for the first time for this archipelago, increasing the recorded Azorean fauna to seven species.

## Introduction

The marine molluscs of the Archipelago of the Azores are probably the best studied marine invertebrate group from these Atlantic Islands. Several taxonomic, ecological, genetic, biogeographic and recent palaeontological studies have greatly improved our knowledge of this phylum (see Ávila 2005 and Ávila et al. 2000a, 2000b, 2011, 2012 and references therein), with more than 11% endemic species (Ávila et al. 2009). Amongst marine molluscs, trochid and rissoid gastropods (Ávila et al. 2011, 2012) and opisthobranchs (Pedro et al. 2011, Cordeiro et al. 2013) were recently given attention; in contrast, few studies deal specifically with chitons: Kaas and Van Belle (1981, 1985a, 1985b), Kaas (1985, 1991), and the more recent paper by Ávila and Albergaria (2002).

Polyplacophorans (or “chitons”) include over 900 extant species worldwide that mostly live in shallow waters, usually on rocky substrates. They are oval in shape and dorso-ventrally flattened, neither tentacles nor eyes are present in the head region, and they possess eight distinctive overlapping shell plates or valves located on the dorsal side. These longitudinally arranged valves are surrounded by a muscular girdle, and the girdle covering or perinotum is ornamented with scales, spicules, bristles or other protuberances (Kaas and Van Belle 1985a; Schwabe 2010). Identification of chitons mainly depends on microscopic feature of the girdle and valves; species are differentiated by patterns in the raised sculpture of the dorsal surface of the valves (tegmentum), and the shape, size, and density of spicular processes of the perinotum. Additional important features are the radula (only visible via dissection) and gills (visible under magnification, externally on the ventral surface between the foot and the girdle).

The present work is based on a review of new material collected from all the islands of the Azores, and updates the published information, documenting for the first time the occurrence of the polyplacophoran molluscs *Lepidochitona* cf. *canariensis* (Thiele, 1909) and *Tonicella rubra* (Linnaeus, 1767) in these oceanic islands.

## Materials and methods

A bibliographic review of the polyplacophoran species reported from the Atlantic Ocean was assembled. More than 1,060 lots from the marine mollusc reference collection of the Department of Biology of the University of the Azores (DBUA – São Miguel Island), corresponding to approx. 850 dives in all islands, were examined and the polyplacophorans sorted and identified from 63 lots (303 specimens and 5 valves; see Table 1). The mollusc collection of the Department of Oceanography and Fisheries of the University of the Azores (DOP/ML – Faial Island) was also surveyed for chitons. Specimens were studied using either a Nikon SMZ 1000 or a Leica M125 stereomicroscope with incandescent light sources (Volpi Intralux 4100) and digital camera attached, which fed images to a desktop computer. The taxonomic organization of species in this list follows the morphological systematics of Sirenko (2006), which is largely in agreement with molecular evidence (Okusu et al. 2003).

**Table 1.** Number of the sampling sites in the Reference Collection of the Department of Biology of the University of the Azores (DBUA) that yielded polyplacophorans, island and location of the sampling sites, depth range (m), number of Specimens (N. spc.), number of valves (N. valv.) and date.<br/>

**DBUA**	**Island / seamount**	**Location**	**Depth range**	**N. spc.**	**N. valv.**	**Date**
176	São Miguel	Ponta da Pirâmide	13 m	1	-	July-1988
190	Flores	Fajã Grande	intertidal zone	3	-	10-July-1989
191	Flores	Ponta Delgada	intertidal zone	1	-	July-1989
193	Flores	Santa Cruz (pool)	intertidal zone	1	-	09-July-1989
240	Flores	Santa Cruz	20 m	2	-	July-1989
332	Formigas Islets	West Bay	6–8 m	1	-	08-June-1990
337	Formigas Islets	Formigas	intertidal zone	7	-	06-June-1990
355	Formigas Islets	Formigas	15 m	-	1	03-July-1991
356	Formigas Islets	Formigas	intertidal zone	1	-	01-July-1991
410	Faial	Ilhéu Negro	10 m	1	-	24-July-1989
433	Faial	Baía de entre-os-montes	?	1	-	26-July-1989
457	Pico	Lajes do Pico	intertidal zone	1	-	July-1989
458	Pico	Lajes do Pico	intertidal zone	2	-	July-1989
459	Pico	Lajes do Pico	intertidal zone	1	-	July-1989
461	Pico	Lajes do Pico	intertidal zone	1	-	July-1989
465	Pico	Lajes do Pico	intertidal zone	-	2	July-1989
475	Pico	Lajes do Pico	intertidal zone	-	1	July-1989
486	Pico	Lajes do Pico	2–4 m	1	-	July-1989
524	Flores	Baixa da Calheta	intertidal zone	1	-	28-Oct-1990
551	Flores	Porto da Baleia	?	1	-	29-Oct-1990
554	Flores	Pontinhas	intertidal zone	6	-	28-Oct-1990
562	Flores	Pontinhas	intertidal zone	17	-	28-Oct-1990
569	Flores	Baixa do Porto	?	1	-	28-Oct-1990
574	Flores	Baixa do Porto	6–12 m	1	-	27-Oct-1990
577	Flores	Lajes das Flores	0–10 m	2	-	27-Oct-1990
625	São Miguel	São Roque	?	2	-	31-May-1991
637	São Miguel	Ponta da Galera	12 m	1	-	July-1989
662	Pico	Lajes do Pico	0–3 m	3	-	19-Aug-1995
667	Pico	Lajes do Pico	0–6 m	5	-	05-Aug-1996
671	Pico	Lajes do Pico	intertidal zone	1	-	18-Aug-1997
683	São Miguel	São Vicente, Capelas	10 m	1	-	02-July-1996
700	São Miguel	São Vicente, Capelas	0–10.4 m	2	-	17-July-1996
708/F	São Miguel	São Vicente, Capelas	8 m	1	-	19-July-1996
715	São Miguel	Cerco, Caloura	intertidal zone	3	-	26-Jan-1996
719	São Miguel	Vila Franca Islet	18 m	-	1	03-Mar-1997
721	São Miguel	Rosto do Cão Islet	15 m	1	-	15-May-1998
725	Flores	Angra do Heroísmo bay	intertidal zone	1	-	15-June-1998
730	São Miguel	Baía da Pranchinha	8.6 m	3	-	04-July-1990
731	São Miguel	Baía da Pranchinha	13.8 m	1	-	04-July-1990
732	São Miguel	Baía da Pranchinha	13.8 m	5	-	04-July-1990
733	São Miguel	Baía do Rosto do Cão	9.5 m	1	-	04-July-1990
740	São Miguel	Baía do Rosto do Cão	intertidal zone	28	-	26-June-1990
741	São Miguel	Baía do Rosto do Cão	intertidal zone	8	-	06-July-1990
743	São Miguel	Baía do Rosto do Cão	intertidal zone	1	-	06-July-1990
744	São Miguel	Baía do Rosto do Cão	intertidal zone	87	-	06-July-1990
745	São Miguel	Baía do Rosto do Cão	intertidal zone	53	-	06-July-1990
746	São Miguel	Baía do Rosto do Cão	intertidal zone	16	-	06-July-1990
747	São Miguel	Baía do Rosto do Cão	intertidal zone	2	-	06-July-1990
748	São Miguel	Capelas	14 m	2	-	07-Oct-1996
751	São Miguel	Baía do Rosto do Cão	subtidal	1	-	05-May-1994
752	São Miguel	Fenais da Luz	intertidal zone	2	-	15-Apr-1992
767	São Miguel	São Vicente, Capelas	20.3 m	1	-	11-July-1997
793	São Miguel	São Vicente, Capelas	intertidal zone	1	-	19-July-1997
794	São Miguel	Ponta Delgada	?	2	-	20-Nov-1996
799	Flores	Santa Cruz	tide pool	1	-	July-1999
800	Pico	São João	15 m	1	-	July-1999
801	Faial	Monte da Guia	23 m	1	-	23-Aug-1999
803	Faial	?	3–6 m	3	-	Sep-1998
857	Pico	Monte	8–10 m	1	-	05-Aug-2005
858	Pico	Barca, Madalena	10 m	1	-	09-Aug-2005
891	D. João de Castro	seamount	20 m	1	-	31-Aug-2004
1047	Pico	Ribeiras harbour	4 m	1	-	07-Aug-2000
1056	São Miguel	Rosto do Cão Islet	intertidal zone	4	-	26-Mar-2012

## Results

### Systematic Part Order LEPIDOPLEURIDA Thiele, 1909 Family HANLEYIDAE Bergenhayn, 1955

Animals ovate to elongate. Sculpture of the tegmentum varying from almost smooth to granular. Spicules and longer spines are present in the perinotum (Kaas and Van Belle 1985a). Unslit insertion plates on head valves, in some species also unslit insertion plates on tail valve and intermediate valves.

#### Genus *Hanleya* Gray, 1857

Tegmentum granulated. Overall, with the characteristics of the family.

##### Hanleya hanleyi

(Bean in Thorpe, 1844)

http://species-id.net/wiki/Hanleya_hanleyi

Figs 1–2

Hanleya debilis Gray, 1857 =
Lepidopleurus carinatus Dall, 1927 =
Hanleya dalli Kaas, 1957 =

###### Records for the area.

Van Belle (1984), Kaas and Van Belle (1985a), Ávila and Albergaria (2002).

###### Distribution and biotope.

From the Barents Sea south to Algarve (Portugal), the Mediterranean Sea, Azores, Madeira, Canary Islands (Kaas 1991), near Iceland, Faroes Islands (Sneli et al. 2005), Greenland and the east coast of North America (Kaas and Van Belle 1985a). It lives from 15 to 555 meters depth (Kaas and Van Belle 1985a). Usually found feeding on coralline algae.

###### Material examined.

Flores (Porto da Baleia: DBUA 551, 1 spm), Pico (Lajes do Pico, 0–3m: DBUA 662, 1 spm).

###### Fossil record.

No fossil representatives are known from the Azores.

###### Description (abridged).

Small (up to 22 × 13 mm), elongate oval, dorsal elevation ratio (intermediate valve height / valve width) up to ~0.3. Valves thick, not beaked and girdle narrow, with spicules. Intermediate valves rectangular. Tegmentum uniformly creamy white to light tan, occasionally with brown mineral deposits; uniformly sculpted with numerous large granules, arranged randomly on the lateral areas of the intermediate valves. Jugal (central) area distinct, with fine longitudinal riblets larger and more widely spaced than those in the pleural (outer) areas. Girdle perinotum covered in randomly distributed projecting spicules.

###### Remarks.

This species is very rare in the Azores.

### Order CHITONIDA Thiele, 1909 Family CALLOCHITONIDAE Plate, 1901

Small to large in size [up to 110 × 80 mm – *Eudoxochiton nobilis* (Gray, 1843)], oval, tegmentum with fine or no apparent granular sculpture, valves appear smooth but with neat rows of black, pigmented shell eyes. Terminal valves multi-slitted (large numbers of insertion teeth), intermediate valves with 1–4 slits on insertion plates. Perinotum with small spicules.

#### Genus *Callochiton* Gray, 1847

Small to medium size [up to 55 × 36 mm – *Callochiton dentatus* (Spengler, 1797)]; extra-pigmentary eyes present. Overall, with the characteristics of the family.

##### Callochiton septemvalvis

(Montagu, 1803)

http://species-id.net/wiki/Callochiton_septemvalvis

Chiton achatinus Brown, 1827 =
Chiton doriae Capellini, 1859 =
Chiton laevis var. *navicula* Jeffreys, 1865 =
Callochiton achatinus euboecus Kattoulas, Koukouras and Economidis, 1973 =
Chiton scytodesma Scacchi, 1836 ?
Chiton laevis Pennant, 1777 *sensu*Montagu 1803 !

###### Records for the area.

Morton (1967), Kaas and Van Belle (1985a).

###### Distribution and biotope.

All Atlantic coasts of Europe, from Scandinavia, Britain and Ireland, south to the Mediterranean Sea, Morocco (Kaas 1991), Azores and Canary Islands (Kaas and Van Belle 1985b). From shallow subtidal to 500 m depth, usually on red algae and other hard substrates (Poppe and Goto 1991). The animals can be extremely cryptic, grazing on the underside of stones and small boulders where their colours proved good camouflage.

###### Material examined.

No material seen.

###### Fossil record.

No fossil representatives are known from the Azores.

###### Description (abridged).

Moderate size (up to 22 × 14 mm), dorsal elevation ratio = 0.35 to 0.46, oval, valves beaked. Tegmentum very finely granulose, orange to brick red, often with white markings, or with shades of green, bright yellow, or bright orange. Intermediate valves rectangular. Sculpture smooth and glossy to the naked eye, diagonally set with black dots (the pigment cups of the ‘shell-eyes’); under magnification the valves are sculptured with small granules arranged in quincunx. Wide girdle, usually about 1/3 of the animal’s total width and covered in spicules, with a short marginal fringe of spicules. The girdle is coloured yellow or orange with red markings.

###### Remarks.

If this species does occur in the Azores, it must be very rare, as not a single specimen was found in the DBUA or DOP/ML collections. The species *Callochiton septemvalvis* was originally described from an abnormal specimen with seven valves. Montagu (1803) believed its missing valve to be a characteristic of an undescribed species of chiton. Although that specimen did represent an undescribed species, normal individuals of *Callochiton septemvalvis* have eight valves. Some authorities have criticised the name ‘ *septemvalvis*’ as being misleading, but as it was the first epithet used to describe a valid species, the name remains valid.

### Family TONICELLIDAE Simroth, 1894

Small to medium in size [up to 55 × 36 mm – *Tonicella insignis* (Reeve, 1847)], oval to elongate oval. Valve tegmentum appears smooth or granulose but without separated sculpture elements, terminal valves with multi-slitted insertion plates, intermediate valves with usually one insertion slit on each side. Girdle perinotum covered in small spicules.

#### Genus *Lepidochitona* Gray, 1821

Oval to elongate-oval, valve sculpture smooth to uniformly granular, girdle perinotum with irregular granules, most species with a short marginal fringe of blunt spicules.

##### Lepidochitona cf. canariensis

(Thiele, 1909)

Figs 3–4

Trachydermon canariensis Thiele, 1909

###### Records for the area.

This is the first record for the Azores.

###### Distribution and biotope.

The Mediterranean Sea (Dell’Angelo and Tringali 2000), Morocco (Dell’Angelo and Smriglio 1999), Madeira, Canary Islands and Cape Verde (Kaas and Van Belle 1985b), Savage Islands (Ilhas Selvagens) (Albuquerque et al. 2009) to Mauritania (Anseeuw and Verstraeten 2009), and now the Azores (this work). Intertidal down to 20 m depth.

###### Material examined.

Formigas Islets (intertidal zone: DBUA 337, 2 spm; DBUA 356, 1 spm), São Miguel (intertidal: DBUA 747, 1 spm).

###### Fossil record.

No fossil representatives are known from the Azores.

###### Description (abridged).

Small size (up to 8.5 × 5 mm), dorsal elevation ratio = 0.39 (Kaas and Van Belle 1985b). Tegmentum sculptured with diamond-shaped granules. Girdle narrow, densely covered in small calcareous pustules and scattered spines.

###### Remarks.

This is a rare species, known only from a small number of specimens in the Azores.

**Figures 1–9.**
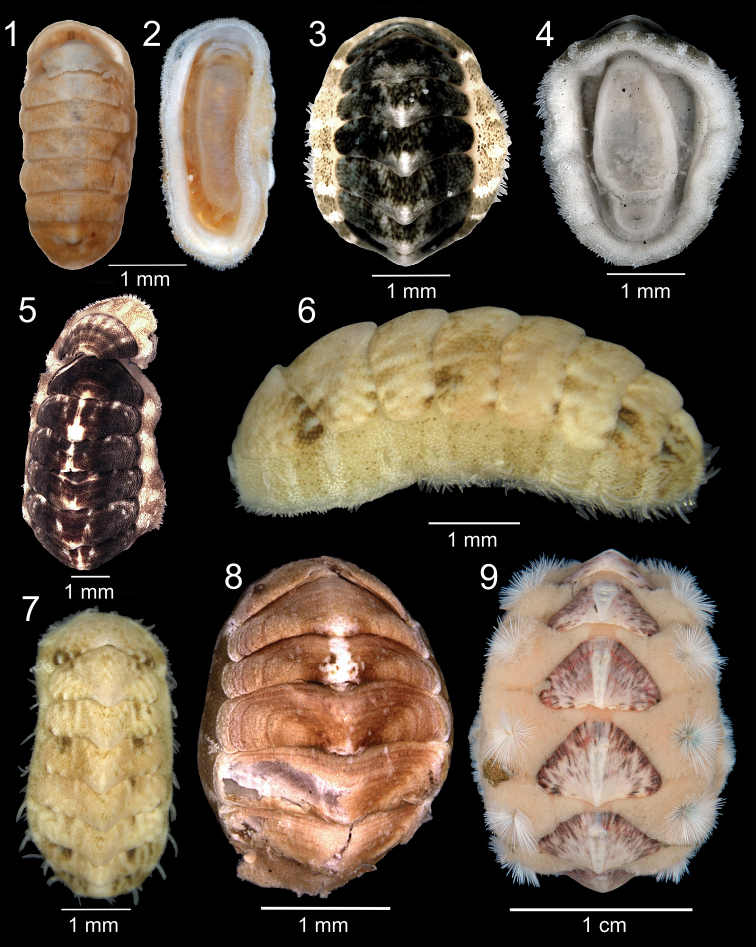
Shallow polyplacophorans from the Azores. **1–2** *Hanleya hanleyi* (Bean in Thorpe, 1844), DBUA 551 (Flores, Porto da Baleia) **3–4** *Lepidochitona* cf. *canariensis* (Thiele, 1909), DBUA 356 (Formigas Islets, intertidal) **5** *Lepidochitona piceola* (Shuttleworth, 1853), DBUA 743 (São Miguel Island, Baía do Rosto do Cão, intertidal) **6–7** *Lepidochitona simrothi* (Thiele, 1902), DBUA 459 (Pico, Lajes do Pico, intertidal) **8** *Tonicella rubra* (Linnaeus, 1767), DBUA 891 (D. João de Castro seamount, 20 m depth) **9** *Acanthochitona fascicularis* (Linnaeus, 1767), DBUA 667 (Pico, Lajes do Pico, 0–6 m depth).

##### Lepidochitona piceola

(Shuttleworth, 1853)

http://species-id.net/wiki/Lepidochitona_piceola

Fig. 5

Chiton (Acanthopleura) piceolus Shuttleworth, 1853
Nuttalina piceolus Pilsbry, 1894
Nuttalina piceola Nierstrasz, 1906
Middendorfia piceola Bergenhayn, 1931

###### Records for the area.

Kaas and Van Belle (1981, 1985b), Van Belle (1984), Kaas (1991).

###### Distribution and biotope.

Azores and Canary Islands (Kaas and Van Belle 1985b). Intertidal.

###### Material examined.

São Miguel (intertidal zone down to 14 m depth: DBUA 625, 1 spm; DBUA 743, 1 spm; DBUA 744, 23 spm; DBUA 745, 14 spm; DBUA 1056, 1 spm).

###### Fossil record.

No fossil representatives are known from the Azores.

###### Description (abridged).

Small size (up to 10 × 6 mm), dorsal elevation ratio = 0.31 (Kaas and Van Belle 1985b). Tegmentum with round quincuncially arranged granules, but valves generally strongly eroded, and sculpture usually preserved only along the anterior margins of the valves. Valves rather thick, with a strong apical callus. Girdle wide, approx. 40% of the total width.

###### Remarks.

This species has been overlooked and confused with *Lepidochitona simrothi*. However, it is easy to separate these two species, as *Lepidochitona piceola* does not have the long, smooth, curved needles characteristic of the girdle of *Lepidochitona simrothi*. The only records before this work were those of Kaas and Van Belle (1985b) who reported the species from the intertidal of São Miguel Island, and Kaas (1991) who reported the species from the Formigas Islets (0-15 m depth).

##### Lepidochitona simrothi

(Thiele, 1902)

http://species-id.net/wiki/Lepidochitona_simrothi

Figs 6–7

###### Records for the area.

Kaas and Van Belle (1981, 1985b), Van Belle (1984), Kaas (1991), Bullock (1995), Morton et al. (1998), Macedo et al. (1999), Ávila et al. (2000a, b), Ávila and Albergaria (2002).

###### Distribution and biotope.

Azores (Kaas and Van Belle 1981; Ávila and Albergaria 2002) and Portugal (Zalvide et al. 2000). Littoral and sublittoral to 14 m depth.

###### Material examined.

Faial (3–6 m depth: DBUA 803, 3 spm), Flores (all samples collected in the intertidal zone: DBUA 190, 3 spm; DBUA 191, 1 spm; DBUA 193, 1 spm; DBUA 524, 1 spm; DBUA 554, 6 spm; DBUA 562, 17 spm), Formigas Islets (intertidal zone: DBUA 337, 5 spm; DOP/ML 0032, 1 spm), Pico (0–3 m depth: DBUA 457, 1 spm; DBUA 458, 2 spm; DBUA 459, 1 spm; DBUA 461, 1 spm; DBUA 465, 2 valves; DBUA 475, 1 valve; DBUA 662, 2 spm); São Miguel (intertidal zone down to 14 m depth: DBUA 625, 1 spm; DBUA 715, 3 spm; DBUA 732, 3 spm; DBUA 740, 28 spm; DBUA 741, 8 spm; DBUA 744, 64 spm; DBUA 745, 39 spm; DBUA 746, 16 spm; DBUA 747, 1 spm; DBUA 793, 1 spm).

###### Fossil record.

No fossil representatives are known from the Azores.

###### Description (abridged).

Animal rather small (up to 8 × 4 mm), dorsal elevation ratio = 0.37 (Kaas and Van Belle 1985b), elongate oval in outline. Tegmentum with round granules. Tail valve very small. Girdle densely covered with small calcareous pustules and distinctive curved spines randomly scattered throughout perinotum armature.

###### Remarks.

Specimens recorded by Hawkins et al. (1990: 27–28) and Azevedo (1991: 29) probably belong to this species but were not identified at species level. These specimens were not present in the DBUA collection and could not be examined by the authors. The image of *Lepidochitona* sp. (Macedo et al. 1999: 75) represents a specimen of *Lepidochitona simrothi*. This is the most common chiton in the Azores.

#### Genus *Tonicella* Carpenter, 1873

With the characteristics of the family. Valves with ‘spongy’ eaves (i.e. porous, penetrated laterally by large aesthete canals).

##### Tonicella rubra

(Linnaeus, 1767)

http://species-id.net/wiki/Tonicella_rubra

Fig. 8

Chiton laevis Pennant, 1777 =
Chiton minimus Spengler, 1797 =
Chiton incarnatus Reeve, 1848 =
Chiton latus Leach, 1852 =
Chiton ruber var. *oblonga* Jeffreys, 1865 =
Tonicella rubra var. *index* Balch, 1906 =
Tonicella beringensis Jakovleva, 1951 =
Tonicella granulata Jakovleva, 1952 =
Tonicella zotini Jakovleva, 1952 =
Tonicella beringensislucida Sirenko, 1974 =
Chiton cinereus Linnaeus, 1767 *sensu* Fabricius, 1780 !

###### Records for the area.

This is the first record for the Azores.

###### Distribution and biotope.

This species has an Arctic-circumboreal distribution, including the Arctic Ocean (Barents Sea, White Sea, Spitzbergen), the North Pacific (northern Japan), the western North Atlantic as far south as New London (Connecticut) (Kaas and van Belle 1985b) and the eastern Atlantic from Greenland and Scandinavia to Britain and Ireland (Kaas and van Belle 1985b), and now the Azores.

###### Material examined.

Dom João de Castro seamount (20 m depth: DBUA 891, 1 spm).

###### Fossil record.

No fossil representatives are known from the Azores.

###### Description (abridged).

Up to 15 × 9 mm in the North Atlantic; dorsal elevation ratio = 0.29. Valves beaked, girdle narrow. Tegmentum appears smooth, with growth lines clearly visible under magnification. Colour orange to pinkish, generally with small reddish-brown blotches. Girdle relatively narrow and covered by small scales, appearing sandy to the naked eye, coloured like the tegmentum, but sometimes with white or cream markings particularly at the junctures between valves.

###### Remarks.

The Dom João de Castro Bank (Lat 38°13.3’N, Long 26°36.2’W) is a shallow seamount (minimum depth = 13 m) located between the islands of São Miguel and Terceira. The last eruption was in December 1720 when a small island (~1 km long and 150 m high) was formed (Agostinho 1934). This island disappeared within a year and nowadays the seamount is capped by a submarine caldera (300 × 600 m) approx. 40 m deep, with strong hydrothermal activity in vents located at approx.20 m depth (Ávila et al. 2004; Cardigos et al. 2005).

### Family ACANTHOCHITONIDAE Pilsbry, 1893

The broad girdle is covered by coarse spines and partially covers the valves. The valve tegmentum (dorsal aspect) is reduced relative to the articulamentum (ventral part covered by the girdle). Head valve usually with five slits in margin.

#### Genus *Acanthochitona* Gray, 1821

Girdle with large distinct bristles, clumps of long straight spicules, emerging from the girdle at the junctures between the valves on both sides.

##### Acanthochitona fascicularis

(Linnaeus, 1767)

http://species-id.net/wiki/Acanthochitona_fascicularis

Fig. 9

Acanthochites communis Risso, 1826 =
Chiton fascicularis var. *major* Philippi, 1836 =
Chiton fascicularis var. *rubra* Issel, 1870 =
Acanthochites discrepans var. *minorflava* Monterosato, 1878 *nomen nudum* =
Acanthochites hamatus Rochebrune, 1882 =
Anisochiton discrepans var. *elongata* Dautzenberg, 1893 =
Anisochiton discrepans var. *marmorata* Dautzenberg, 1893 =
Anisochiton discrepans var. *nigrolineata* Dautzenberg, 1893 =
Acanthochites discrepans var. *albina* Dautzenberg and Durouchoux, 1900 =
Anisochiton discrepans var. *viridis* Pallary, 1902 =
Acanthochites discrepans var. *violaceolimbata* Dautzenberg and Durouchoux, 1906 =
Acanthochiton discrepans var. *angustivalva* Bergenhayn, 1931 =
Acanthochiton heterochaetus Bergenhayn, 1931 =
Acanthochiton communis var. *barashi* Leloup, 1969 =
Acanthochitona bonairensis Kaas, 1972 =
Chiton echinotus de Blainville, 1825 ?
Acanthochites carinatus Risso, 1826 ?
Chiton crinitus Pennant, 1777 *sensu* Sowerby, G.B. II, 1840 !
Chiton discrepans Brown, 1827 *sensu* Sowerby, G.B. II, 1840 !

###### Records for the area.

MacAndrew (1856), Dautzenberg (1927), Morton (1967), Van Belle (1984), Kaas (1985, 1991), Ávila and Albergaria (2002).

###### Distribution and biotope.

Found in the North Atlantic from Ireland and Britain, south to Portuguese shores (Nobre 1931), Azores, Madeira, Selvagens (Albuquerque et al. 2009), Canary Islands and throughout the Mediterranean Sea (Kaas 1985, 1991). From the intertidal zone down to 50 m depth (Van Belle 1984).

###### Material examined.

Faial (10–23 m depth: DBUA 410, 1 spm; DBUA 433, 1 spm; DBUA 801, 1 spm), Flores (intertidal zone down to 20 m depth: DBUA 240, 2 spm; DBUA 569, 1 spm; DBUA 574, 1 spm; DBUA 577, 2 spm; DBUA 725, 1 spm; DBUA 799, 1 spm), Formigas (6–15 m depth: DBUA 332, 1 spm; DBUA 355, 1 valve), Pico (intertidal zone down to 15 m depth: DBUA 486, 1 spm; DBUA 667, 5 spm; DBUA 671, 1 spm; DBUA 800, 1 spm; DBUA 857/DOP/ML 0050, 1 spm; DBUA 858/DOP/ML0051, 1 spm; DBUA 1047, 1 spm), São Miguel (intertidal zone down to 20 m depth: DBUA 176, 1 spm; DBUA 637, 1 spm; DBUA 683, 1 spm; DBUA 700, 2 spm; DBUA 708/F, 1 spm; DBUA 719, 1 valve; DBUA 721, 1 spm; DBUA 730, 3 spm; DBUA 731, 1 spm; DBUA 732, 2 spm; DBUA 733, 1 spm; DBUA 748, 2 spm; DBUA 751, 1 spm; DBUA 752, 2 spm; DBUA 767, 1 spm; DBUA 794, 2 spm; DBUA 1056, 3 spm).

###### Fossil record.

No fossil representatives are known from the Azores.

###### Description (abridged).

Rather large, up to 24 × 15 mm (DBUA 667). Overall colour is variable (black, blue, olive, orange, cream). Valves typically olive-green with reddish blotches; the wide jugal area is usually lighter (cream or beige) with dark longitudinal streaks. Visible portion of the valves is trapezoidal, little or no beak. The central area with faint longitudinal grooves; lateral areas covered with closely-spaced, small round granules in quincunx and forming radiating rows. Girdle broad and densely covered with short spicules, with 18 large tufts of spines arranged around the head and at the sutures. One specimen from the Azores had a girdle that appeared cyan blue in life. There is a distinct marginal fringe of small tapered spicules.

###### Remarks.

This is the largest chiton in the Azores, commonly found under stones buried in pebbles or in sand, in shallow water, sometimes in groups of two or three specimens. *Acanthochitona fascicularis* is particularly variable in colour.

## Discussion

Van Belle (1984) enumerated eight species of chitons from the Azores: *Hanleya hanleyi*, *Lepidochitona piceola*, *Lepidochitona simrothi*, *Ischnochiton albus* [= *Stenosemus albus* (Linnaeus, 1767)], *Ischnochiton exaratus* [= *Stenosemus exaratus* (Sars, 1878)], *Placophoropsis atlantica* [= *Placiphorella atlantica* (Verrill & Smith in Verrill, 1882], *Acanthochitona fascicularis* and *Acanthochitona communis* (Risso, 1826) [= *Acanthochitona fascicularis* (Linnaeus, 1767)]. Of these, one was a synonym of *Acanthochitona fascicularis*, and three were deep-water species (*Stenosemus exaratus*, *Stenosemus albus* and *Placiphorella atlantica*); therefore only four shallow-water species of chitons were reported from the Azores by this author. Ávila and Albergaria (2002) reported five species of Polyplacophora from the Azores and considered *Acanthochitona discrepans* (Brown, 1827), reported by MacAndrew (1856: 145), Dautzenberg (1889: 127) and Nobre (1924: 84; 1930: 61) but not cited by Van Belle (1984) as “highly questionable”. No specimens of this species were found in this survey, so its status remains as a doubtful record. The presence of *Callochiton septemvalvis* is based on a single historical observational record by Morton (1967) and no preserved specimens are known from the Azores. Nevertheless, we tentatively accept this record as likely since *Callochiton septemvalvis* is widely distributed in the North Atlantic but highly cryptic, living in the very low intertidal to 160 m, and at low population densities (Jones and Baxter 1987). Kaas (1985: 580) reported *Acanthochitona crinita* (Pennant, 1777) from the Azores [(it exists) “from (…) Norway, S to the Cape Verde Archipelago”], but the same author unequivocally stated that this species is “not (present) in the Azores” (Kaas, 1991: 95). Notwithstanding recent reports from the area (Segers et al. 2009, Rolán 2011, Moreno and Gofas 2011), which are based solely on bibliographic records, we disregard this species as occurring in the archipelago and suggest that it should be eliminated from the Azores shallow-water marine mollusc checklist. Thus, the recorded Azorean shallow-water polyplacophoran fauna consists of seven living species, as no fossil chitons are known from the Azores. Four species (*Hanleya hanleyi*, *Callochiton septemvalvis*, *Tonicella rubra*, and *Acanthochitona fascicularis*) are common to the north-east Atlantic, but the ubiquitous northern European species *Lepidochitona cinerea* (Linnaeus, 1878) is absent and replaced by three other warmer-water species of the same genus. The summary presented here includes the first Azorean records of two species: *Lepidochitona* cf. *canariensis* and *Tonicella rubra*. The discovery of *Tonicella rubra* on a shallow, small (~18 ha area of summit) and young seamount (~300 y) leads us to believe that the chitons of the Azores are still poorly known when compared with other molluscan classes.

## Supplementary Material

XML Treatment for Hanleya hanleyi

XML Treatment for Callochiton septemvalvis

XML Treatment for Lepidochitona cf. canariensis

XML Treatment for Lepidochitona piceola

XML Treatment for Lepidochitona simrothi

XML Treatment for Tonicella rubra

XML Treatment for Acanthochitona fascicularis
